# The histone H3 variant H3.3 regulates gene body DNA methylation in *Arabidopsis thaliana*

**DOI:** 10.1186/s13059-017-1221-3

**Published:** 2017-05-18

**Authors:** Heike Wollmann, Hume Stroud, Ramesh Yelagandula, Yoshiaki Tarutani, Danhua Jiang, Li Jing, Bhagyshree Jamge, Hidenori Takeuchi, Sarah Holec, Xin Nie, Tetsuji Kakutani, Steven E. Jacobsen, Frédéric Berger

**Affiliations:** 1 Temasek Lifesciences Laboratory, 1 Research Link, National University of Singapore, Singapore, 117604 Singapore; 2 Present address: Institute of Molecular and Cell Biology, 61 Biopolis Drive, Proteos, Singapore, 138673 Singapore; 3 Department of Molecular, Cell, and Developmental Biology, University of California, Los Angeles, CA 90095 USA; 4 Howard Hughes Medical Institute, University of California, Los Angeles, CA 90095 USA; 5 Department of Integrated Genetics, National Institute of Genetics, Shizuoka, 411-8540 Japan; 6 Department of Genetics, School of Life Science, The Graduate University for Advanced Studies (SOKENDAI), Shizuoka, 411-8540 Japan; 7 Gregor Mendel Institute, Vienna Biocenter VBC, Dr. Bohr-Gasse 3, 1030 Vienna, Austria; 8 College of Life Science and Technology, Huazhong Agricultural University, No.1, Shizishan Street, Hongshan District Wuhan, Hubei, 430070 China; 9 Department of Biological Sciences, Graduate School of Science, The University of Tokyo, Hongo, Bunkyo-ku, Tokyo, 113-0033 Japan; 10 Eli and Edythe Broad Center of Regenerative Medicine and Stem Cell Research, University of California, Los Angeles, CA 90095 USA

**Keywords:** H3.3, Histone variants, H2A.Z, Linker histone H1, DNA methylation, Chromatin

## Abstract

**Background:**

Gene bodies of vertebrates and flowering plants are occupied by the histone variant H3.3 and DNA methylation. The origin and significance of these profiles remain largely unknown. DNA methylation and H3.3 enrichment profiles over gene bodies are correlated and both have a similar dependence on gene transcription levels. This suggests a mechanistic link between H3.3 and gene body methylation.

**Results:**

We engineered an H3.3 knockdown in *Arabidopsis thaliana* and observed transcription reduction that predominantly affects genes responsive to environmental cues. When H3.3 levels are reduced, gene bodies show a loss of DNA methylation correlated with transcription levels. To study the origin of changes in DNA methylation profiles when H3.3 levels are reduced, we examined genome-wide distributions of several histone H3 marks, H2A.Z, and linker histone H1. We report that in the absence of H3.3, H1 distribution increases in gene bodies in a transcription-dependent manner.

**Conclusions:**

We propose that H3.3 prevents recruitment of H1, inhibiting H1’s promotion of chromatin folding that restricts access to DNA methyltransferases responsible for gene body methylation. Thus, gene body methylation is likely shaped by H3.3 dynamics in conjunction with transcriptional activity.

**Electronic supplementary material:**

The online version of this article (doi:10.1186/s13059-017-1221-3) contains supplementary material, which is available to authorized users.

## Background

Two major types of histone H3 variants evolved in multicellular eukaryotes, H3.1 and H3.3, distinguished by a few amino acid residues as well as their expression patterns and modes of deposition by distinct chaperones [1–3]. While H3.1 expression is coupled to DNA replication, H3.3 expression occurs throughout the cell cycle [4]. H3.3 is a crucial chromatin component required for development in *Drosophila* [5, 6], mouse [7], and *Xenopus* [8]. Notably H3.3 and HIRA are required for reprogramming events during development in animals [9–13] and plants [14, 15].

H3.3 is associated with actively expressed genes in both animals and plants [4, 16–19]. More specifically, genome-wide analysis of chromatin immunoprecipitation (ChIP) in several model organisms, including plants, showed that H3.3 is predominantly enriched near transcription end sites (TES) of genes and positively associated with transcription [18–21], suggesting a direct mechanistic link between H3.3 enrichment and transcription. This distinctive pattern of H3.3 over genes overlaps with the enrichment of RNA polymerase II (RNAPII) [19, 21]. However, H3.3 knockdown has a limited impact on transcription in *Drosophila* [5] and mouse embryonic stem cells (mESCs) [22]. Thus, the functional relationship between H3.3 enrichment and transcriptional activity remains unresolved.

Transcriptional activity has also been related to DNA methylation on gene bodies in mammals, *Arabidopsis*, and other plants [23–25]. In mammals, gene body methylation is maintained by the recruitment of DNA methyltransferase by H3K36me3 [26]. In mammals, H3.3K36me3 is positively correlated with transcriptional activity, elongation, and splicing [27], thus providing a potential mechanism to explain the link between transcription and gene body methylation. However, the prevalence and overall shape of the profile of gene body methylation observed in mammals is not present in other animal groups. In the plant lineage, gene body methylation is largely absent in green algae and bryophytes but is present in most vascular plants [28–31]. In *Arabidopsis*, the similarity between the profiles of gene body methylation and enrichment of H3.3 suggests a link but the mechanism involved remains unknown. To investigate this question, we engineered *Arabidopsis* lines deficient in H3.3 and report decreased gene body methylation in these lines. We further identify that H3K36 methylation and other transcription-related H3 modifications do not play a role in gene body methylation. Instead, we show that H3.3 prevents the deposition of the linker histone H1 on gene bodies, and relaxes chromatin in correlation with transcriptional activity. We propose that this action of H3.3 promotes access to DNA methyltransferase and explains the origin of the transcription-dependent profile of gene body methylation in *Arabidopsis*.

## Results

### H3.3 impacts plant development

In *Arabidopsis* H3.3 is encoded by three *HISTONE 3 RELATED* (*HTR*) genes, *HTR4* (At4g40030), *HTR5* (At4g40040), and *HTR8* (At5g10980), which are highly expressed throughout development [14, 32]. To obtain a knockout line devoid of *H3.3* genes we combined T-DNA insertion lines to generate the double mutant combinations *htr4/htr8* and *htr5/htr8*, which were phenotypically normal despite the absence of the respective full length transcripts (Additional file 1: Table S1; Additional file 2: Figure S1a, b). To obtain a complete H3.3 knockout mutant, we designed a CRISPR/Cas9-based approach to functionally delete both HTR4 and HTR5 (Additional file 1: Tables S1 and S2; Additional file 2: Figure S1c). *htr4;htr5* double homozygous mutants were crossed to *htr8*; however, we were not able to isolate triple homozygous plants (Additional file 2: Figure S1d). Reciprocal crosses suggested that the knockout of H3.3 impaired male gametogenesis and caused embryo lethality (Additional file 2: Figure S1d). We concluded that it would be impossible to obtain somatic tissues completely devoid of H3.3 using this strategy.

**Fig. 1:**
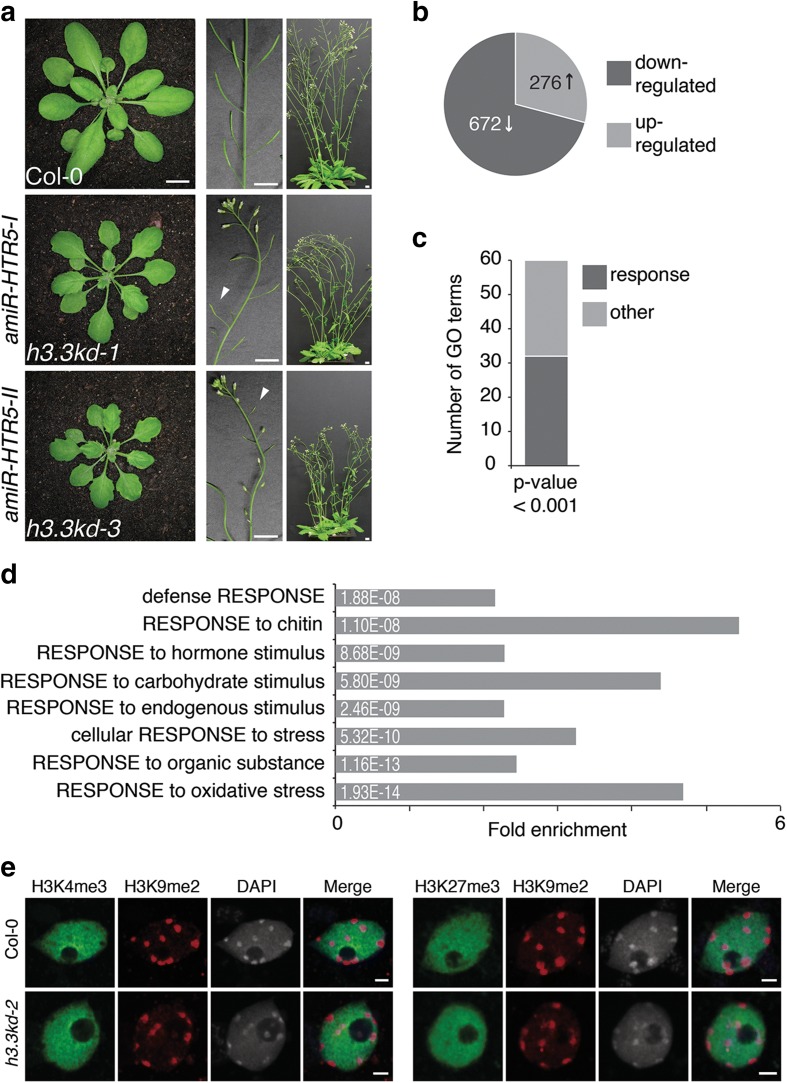
Knockdown of H3.3 causes various phenotypic defects and misregulation of response genes. **a** The impact of H3.3 knockdown on plant growth and development includes serrated leaf shape and smaller rosette size (*left panel*), partial sterility (*middle panel*) and reduced height (*right panel*) of flowering transgenic *h3.3kd* plants compared to wild type (*Col-0*). WT control plants (*top row*) are shown with two independent transgenic lines, both double homozygous for *htr4* and *htr8* (*htr4/htr8*) alleles with either *pHTR5-amiR-HTR5-I* (*middle row*; *h3.3kd-1*) or *pHTR5-amiR-HTR5-II* (*bottom row*; *h3.3kd-3*) artificial miRNAs. **b** The total number of significantly up- and downregulated genes in RNA-seq data from *h3.3kd-3* compared to WT plants. **c** Summary of the Gene Ontology (GO) analysis of misregulated genes in *h3.3kd-3* compared to WT. The bar graph represents the number of response related GO terms compared to others with *p* values less than 0.001. **d** Enrichment and *p* values for selected GO terms. The complete list can be found in Additional file 5. **e** Chromatin localization of H3K4me3, H3K27me3, and H3K9me2 in WT Col-0 and *h3.3kd-2* plants as detected by immunofluorescence in nuclei isolated from mature leaves. DAPI staining shown in *grey*

As an alternative, to effectively reduce levels of *H3.3* transcripts in vegetative tissues we combined the alleles *htr4* and *htr8* with artificial microRNAs (amiRNAs) targeting *HTR5* (Additional file 1: Table S2; Additional file 2: Figure S2a). We constructed two amiRNAs (*amiR-HTR5-I/II*) targeting different regions of the *HTR5* transcript and introduced them into plants segregating from *htr4/+;htr8/htr8* plants. In contrast to double homozygous *htr4/htr4;htr8/htr8* plants that looked similar to wild type (WT; Additional file 2: Figure S2b), *htr4/htr4;htr8/htr8* plants that carried either *amiR-HTR5-I* or *amiR-HTR5-II* (collectively referred to as *h3.3kd* lines) showed serration of leaf margins, reduced growth, and partial sterility (Fig. 1a; Additional file 1: Table S2; Additional file 2: Figure S2b, c). Transcriptome analyses by RNA-seq revealed that *HTR5* transcript levels were reduced in *h3.3kd* plants (Additional file 2: Figure S2d). As a control, the introduction of an *amiR-HTR5-II* resistant version (*rH3.3)* into *h3.3kd* led to the partial rescue of the phenotypic defects observed in *h3.3kd* plants (Additional file 1: Table S2; Additional file 2: Figure S2b, c), confirming that H3.3 knockdown was responsible for the morphological defects observed in *h3.3kd* plants. We noted that serrated leaf margins are prominent in mutants for the H3.3 chaperone complex [15]. Transcriptome analyses in *h3.3kd* plants revealed that the reduction of transcript levels of the three *H3.3* genes caused increased levels of three out of five *H3.1* genes, while the expression levels of genes putatively involved in H3 deposition were not significantly misregulated (Additional file 1: Table S3; Additional file 2: Figure S2d). Because we did not observe any phenotypes in plants overexpressing H3.1-GFP [14], it appears unlikely that phenotypes observed in *h3.3kd* plants resulted from the increased expression of H3.1 variants. Overall, the loss of H3.3 dosage relative to the total pool of H3 led to pleiotropic phenotypic defects, while a complete loss of H3.3 caused lethality. Thus, H3.3 is an essential, non-redundant component of plant chromatin.

### Impact of *h3.3kd* on transcription

H3.3 knockdown caused a variety of developmental defects in plants, suggesting transcription misregulation. We assessed the impact of H3.3 knockdown on transcription using RNA-seq analysis. To minimize secondary effects of H3.3 knockdown on transcription from differences in development, we used WT and *h3.3kd* plants at the seedling stage, where phenotypic defects of *h3.3kd* are less severe. Over 900 genes were significantly misexpressed in *h3.3kd* (Fig. 1b; Additional files 3 and 4), with the majority being downregulated. However, the transcriptional changes in *h3.3kd* were not tightly correlated with enrichment of H3.3 in WT over promoters and/or gene bodies (Additional file 1: Table S4). Hence, although there is a clear correlation between transcriptional activity and H3.3 enrichment over bodies of active genes [18, 19] and promoters [17], H3.3 might not be directly required for transcription.

Gene expression is associated with specific chromatin modifications of histone H3. To investigate the global chromatin architecture in *h3.3kd* we performed immunofluorescence staining. Euchromatin marked by H3K4me3 and H3K27me3 and heterochromatin marked by H3K9me2 showed similar patterns in nuclei from WT and *h3.3kd* leaves (Fig. 1e). Thus, global chromatin organization remained intact in *h3.3kd*. H3K4me3 and H3K36me3 accompany transcriptionally active genes [33]. We compared the profiles of these modifications in WT and *h3.3kd* across gene bodies and observed very little impact on the H3K4me3 profile, consistent with the fact that H3.3 is not enriched at the 5′ end of genes (Additional file 2: Figure S3a). However, we found that H3K36me3 profiles were affected by the loss of H3.3 (Additional file 2: Figure S3b). Promoters of genes downregulated in *h3.3kd* versus WT showed reduced levels of H3K36me3 (Additional file 2: Figures S3d). In contrast, upregulation of genes in *h3.3kd* versus WT correlated with elevated H3K36me3 levels at the 5′ end of genes over gene bodies (Additional file 2: Figure S3f). In contrast to H3K36me3, the levels and profiles of H3K4me3 did not accompany changes in transcriptional activity (Additional file 2: Figure S3c, e).

The loss of H3.3 affected transcription of a relatively limited number of genes. Gene ontology (GO) analysis of downregulated genes revealed a large variety of response processes, including environmental and endogenous stimuli (Fig. 1c, d; Additional file 5). In contrast to stably expressed housekeeping genes, responsive genes are typically differentially regulated during development or in response to stress or other stimuli [34]. A comprehensive analysis of a large set of *Arabidopsis* expression data led to the identification of a set of hypervariable genes that most dramatically change expression levels between different tissues or in response to stimuli and housekeeping genes with nearly constant expression [34]. Of the 123 identified hypervariable genes, 44 were downregulated in *h3.3kd*, while only one of the 379 housekeeping genes was affected (*p* = 8.58 e-35, hypergeometric probability; see “Methods” for details). We thus conclude that H3.3 is not required for gene expression in a global manner. Yet, the loss of H3.3 directly or indirectly affects the expression of subsets of particularly dynamic, responsive, and hypervariable genes.

### Loss of DNA methylation in *h3.3kd*

**Fig. 2:**
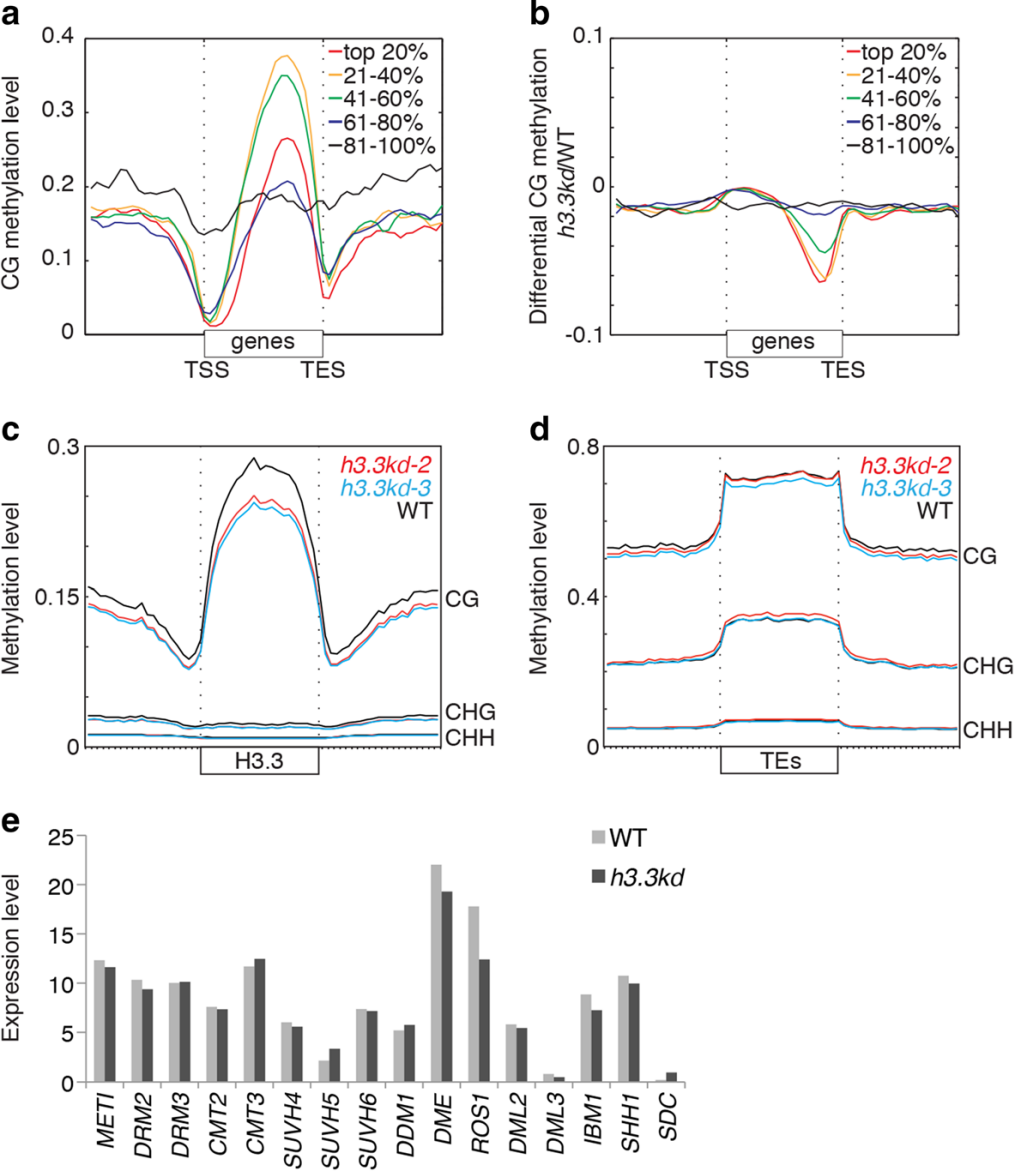
Depletion of DNA methylation over gene bodies and H3.3-enriched regions in *h3.3kd*. **a**–**d** Genome-wide BS-seq results showing enrichment profiles of DNA methylation in WT, *h3.3kd-2*, and *h3.3kd-3*. **a** CG DNA methylation patterns over genes in WT plants. Genes were aligned from transcription start site (*TSS*) to transcription end site (*TES*) and grouped into quintiles according to their level of expression. **b** Relative CG methylation levels over gene bodies in *h3.3kd* compared to WT. Note pronounced loss of methylation at the 3′ ends of highly and moderately expressed genes. **c**, **d** DNA methylation levels in all contexts (CG, CHG, and CHH) over H3.3-enriched regions (**c**) and TEs (**d**) in WT and *h3.3kd*. **e** Expression of DNA methylation-related factors in WT compared to *h3.3kd-3* (RNA-seq)

Many chromatin marks other than H3.3 correlate with gene expression, including DNA methylation. Gene body methylation consists of DNA methylation in CG contexts, that is, is enriched towards the 3′ end of active genes [35–37], partially overlapping with the predominant domain of H3.3 enrichment [18, 19]. The similarities between these two patterns prompted us to investigate the impact of H3.3 depletion on the deposition of DNA methylation over gene bodies using genome-wide sequencing of bisulfite-converted DNA (BS-seq) from mature leaves of WT and *h3.3kd* plants. In agreement with previous reports [35–38], methylation levels over gene bodies increased towards the TES and were highest over expressed genes, but not the most highly expressed genes in WT plants (Fig. 2a). In *h3.3kd* plants we noticed a distinct loss of CG methylation over gene bodies. We identified 16,711 hypomethylated regions (hypo-CG-DMRs) in *h3.3kd*; 89% of hypo-CG-DMRs overlapped with gene bodies and 70% of hypo-CG-DMRs overlapped with H3.3 enriched regions (defined in [18]). Loss of CG methylation over gene bodies was more severe with increasing gene expression level in the WT (Fig. 2b). More generally, DNA methylation levels, normally highly increased over H3.3-enriched regions in WT plants, were reduced in *h3.3kd* (Fig. 2c). In contrast, only 3.1% of TEs in the genome overlapped with hypo-CG-DMRs (Additional file 6), and DNA methylation profiles over transposable elements (TEs) remained largely unaffected in all sequence contexts (Fig. 2d), indicating that the impact of *h3.3kd* on DNA methylation was specific to gene bodies and other H3.3 enriched regions. In conclusion, H3.3 appeared to be required for the deposition or maintenance of DNA methylation over gene bodies.

### The linker histone H1 links H3.3 and DNA methylation

**Fig. 3:**
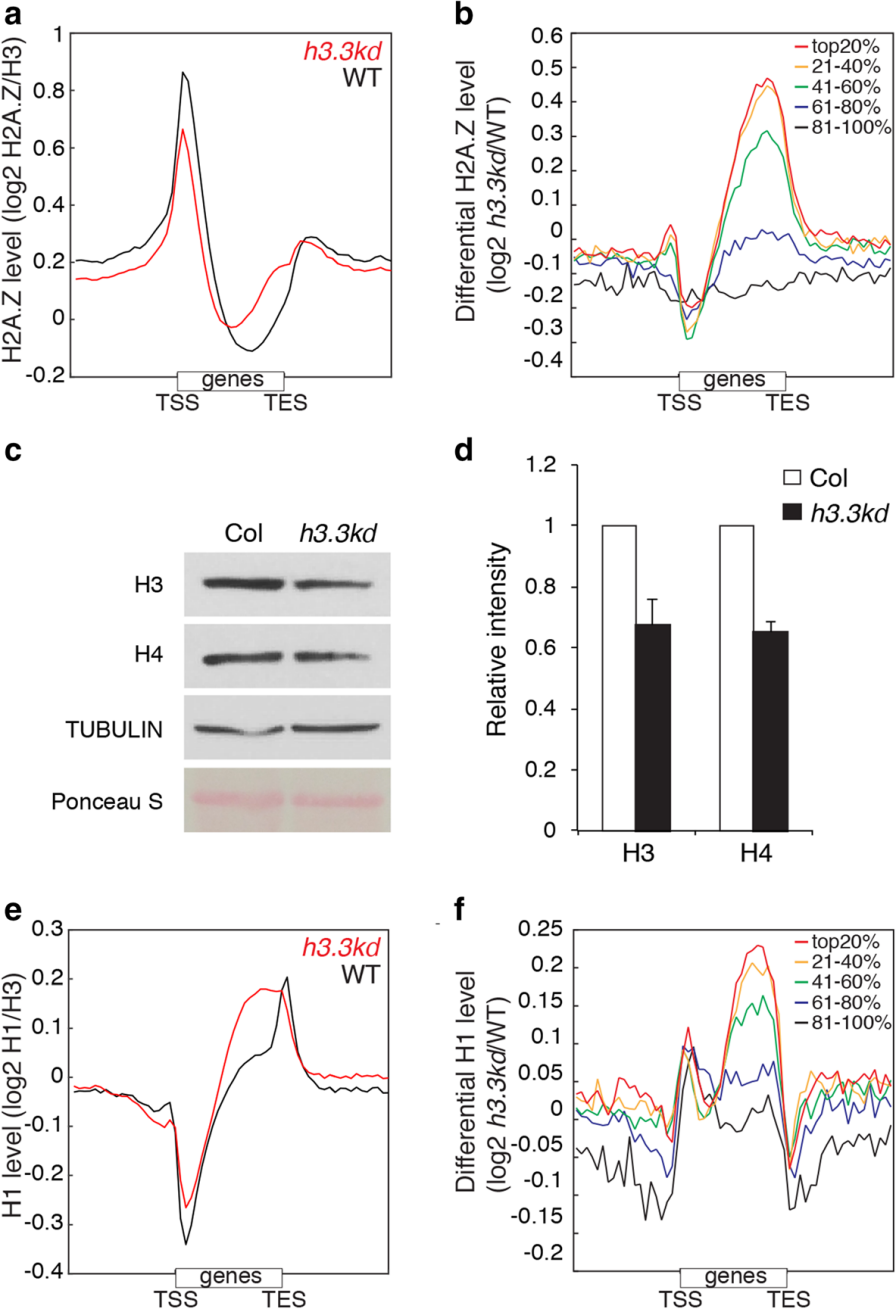
Loss of H3.3 impacts chromatin profiles of H2A.Z and H1. **a**, **b** ChIP-seq profiles depicting the enrichment of H2A.Z in WT and *h3.3kd* over all genes (**a**) and the differential profiles of *h3.3kd* versus WT over genes separated according to their level of expression (**b**). **c** Western blot on total H3 and H4 in Col-0 and *h3.3kd* plants. One of three replicates with similar results is shown. **d** Quantification of H3 and H4 protein abundance in Col-0 and *h3.3kd* plants from western blot analysis in three replicates. Bars represent standard deviation, *n* = 3. **e**, **f** ChIP-seq profiles depicting the enrichment of H1 in WT and *h3.3kd* over all genes (**e**) and the differential profiles of *h3.3kd* versus WT over genes separated according to expression level (**f**)

We searched for a mechanism to explain the loss of DNA methylation over gene bodies in H3.3 knockdown lines. Gene body methylation antagonizes the deposition of the histone variant H2A.Z [39]. We profiled H2A.Z in *h3.3kd* and found that H2A.Z increased over gene bodies in *h3.3kd* compared to WT (Fig. 3a), particularly towards 3′ gene ends where we observed a decrease of DNA methylation (Fig. 2a). This modified profile of H2A.Z in the *h3.3kd* correlated positively with gene expression and was most pronounced over highly expressed genes (Fig. 3b), which also exhibit the strongest levels in H3.3 [18, 19] and DNA methylation. It was reported that H2A.Z does not antagonize DNA methylation [40], leading to the conclusion that H3.3 promotes DNA methylation, which in concert with H3.3 prevents deposition of H2A.Z towards the 3′ end of gene bodies.

The loss of H3.3 had no effect on the expression of DNA demethylases, DNA methyltransferases, and associated factors (Fig. 2e). Thus, the loss of DNA methylation on gene bodies in *h3.3kd* is not likely due to decreased activity of DNA methyltransferases or increased activity of DNA demethylases. The profile of H3K36me3 in WT and *h3.3kd* are not correlated with gene body methylation (Additional file 2: Figure S3), thus supporting that mechanisms involved in gene body methylation in *Arabidopsis* do not rely on this mark, in contrast with mammals. The loss of H3.3 expression caused overexpression of genes encoding H3.1, H2A, H2B, and H4 (Additional file 1: Table S3). The predicted increased levels of core histones could compensate for the deficit of nucleosomes. We thus performed western blotting and observed that total H3 and H4 protein levels were reduced in *h3.3kd* versus WT (Fig. 3c, d). We thus concluded that the depletion of H3.3 results in lower density of nucleosomes over chromatin in general, but likely more pronounced over active genes, where H3.3 is highly enriched in WT plants.

In eukaryotes, the linker histone H1 binds to exposed linker DNA between nucleosomes and promotes chromatin folding [41, 42]. In vitro experiments suggest that H1 deposition into the chromatin is anti-correlated with nucleosome density [43]. In *Arabidopsis* it was proposed that H1 prevents access of DNA methyltransferases to pericentromeric heterochromatin, thus explaining the increase of DNA methylation over these regions in loss of function mutants for H1 [44]. We observed an increased expression of the three genes encoding H1 in *h3.3kd* versus WT (Additional file 1: Table S3), suggesting that H1 density could increase over gene bodies to compensate for the reduction of nucleosome density caused by the loss of H3.3. This hypothesis was supported by a marked invasion of gene bodies by H1 in *h3.3kd* in comparison with WT (Fig. 3e). The change of profile of H1 was correlated positively with gene expression (Fig. 3f) and anti-correlated with the loss of gene body methylation in *h3.3kd* (Fig. 2). These results suggest that H3.3 counters the deposition of H1 over gene bodies. The lack of H3.3 is responsible for the relative enrichment in H1 that opposes DNA methylation over gene bodies in a transcription-dependent manner.

## Discussion

Our study shows that H3.3 is required for transcription of a subset of genes with a marked effect on genes involved in responses to environmental or developmental cues. These genes are subjected to transcriptional reprogramming and harbor a chromatin environment distinct from genes constitutively expressed and involved in basic cell functions. Hence, in *Arabidopsis*, H3.3 loss-of-function affects genes regulating predominantly development and response to the environment but not housekeeping genes. Similarly, loss of H3.3 affects development in *Xenopus*, *Drosophila*, and mouse [6–8], while in transcriptionally stable human embryonic stem cells the impact of H3.3 loss-of-function on gene expression is limited and abnormalities occur only upon their differentiation [22]. H3.3 opposes the deposition of H2A.Z at the 3′ end of gene bodies. H2A.Z also affects expression of hypervariable genes. Nucleosomes containing both H2A.Z and H3.3 are unstable [45] and we propose that these two variants promote the turnover of nucleosomes and likely provide a specific chromatin environment, enabling faster adaptation of the chromatin composition to the requirements of the transcriptional machinery.

Recent analyses have shown that bryophytes and the flowering plant *Eutrema salsugineum* are devoid of gene body methylation, thus questioning its requirement in land plants [28]. Yet, gene body methylation has been implicated in several transcription-related processes, like maintaining the constitutive expression of housekeeping genes, preventing erratic transcription, or enhancing the accuracy of splicing [46]. Our analysis reveals an unexpected link between H3.3 enrichment and gene body DNA methylation.

H3K36me3 has been shown in mammals to facilitate docking of de novo methyltransferase to chromatin [26]. In *Arabidopsis*, however, the profile of H3K36me3 over gene bodies is opposite to that of DNA methylation and H3K36me3 levels over gene bodies increased in *h3.3kd*, specifically at 5′ gene ends, opposite to the domain where DNA methylation is most affected. These observations are incompatible with the idea that H3K36me3 could recruit DNA methyltransferases over gene bodies. This conclusion is also coherent with the absence of a significant change of gene body methylation in the mutant *sdg8* with reduced H3K36me3 [47], suggesting that the mechanisms that recruit DNA methyltransferases to gene bodies are distinct between flowering plants and mammals. It is possible that a specific modification present only or primarily on H3.3 participates in recruiting DNA methyltransferases. The maintenance DNA methyltransferase MET1 contains a bromo adjacent homology domain (BAH) of unknown binding specificity, which might recognize an H3.3-specific mark enriched over gene bodies and H3.3 regions. Yet the identity of this mark remains unknown. Another alternative is the possibility that a mark present only on H3.1 prevents the recruitment of DNA methyltransferases. In *h3.3kd*, ectopic enrichment of H3.1 would then lead to a decrease of gene body methylation.

## Conclusions

We envisage that H3.3 enrichment regulates the recruitment of DNA methyltransferases. The specific profile of H3.3 enrichment over genes is required to retain an adequate density of nucleosomes to maintain suitable chromatin structure for transcription, while providing sufficient accessibility to DNA methyltransferases that methylate gene bodies. When the supply of H3.3 decreases, the linker histone H1 invades gene bodies, while the profile of H1 in promoters does not change significantly. Notably, the anti-correlation of H3.3 and H1 appears to be a conserved mechanism to maintain an open chromatin formation. H3.3 knockdown in *Drosophila* and mouse leads to increased H1 levels [48, 49]. The ability of H1 to promote folding [50] predictably reduces chromatin accessibility, thus preventing access to DNA methyltransferases and reducing gene body methylation. Low gene body methylation levels might then allow ectopic recruitment of H2A.Z containing nucleosomes across the gene bodies. We propose that H3.3 is crucial to safeguard ideal chromatin structure suitable for transcription by maintaining an optimal nucleosome density and preventing H1 deposition over gene bodies. Transcriptional activity promotes H3.3 deposition and DNA methyltransferases maintain the characteristic gene body methylation profile that is positively correlated with gene expression. Gene body methylation prevents deposition of unstable H2A.Z/H3.3 nucleosomes [45, 51], which are generally found at nucleosome-free regions like at promoters. This negative feed-back loop between H3.3, H1, DNA methylation, and H2A.Z might sustain steady transcriptional activity across active genes. This mechanism involves proteins with functions and properties conserved in multicellular eukaryotes, suggesting that it might also play a role in regulating gene body methylation in mammals.

## Methods

### Plant material and growth conditions

All wild-type (WT), mutant, and transgenic lines were in the Columbia-0 (Col-0) ecotype. T-DNA insertion lines were analyzed by PCR-based genotyping and the absence of full-length transcript was confirmed by RT-PCR using primers located in the 5′ and 3′ UTRs. H3.3 T-DNA insertion lines are listed in Additional file 1: Table S1 and primer sequences in Additional file 1: Table S5. For double mutant combinations, lines *htr4-1* (N582765), *htr4-2* (N807939), or *htr5-3* (N846395) were combined with *htr8-2* (N641101). Plants were grown directly on soil in growth rooms with short day (SD) condition for a period of 4–5 weeks and then shifted to long day (LD) condition. Pictures were taken at different growth stages of soil-grown plants. For the methylation analysis, mature leaves of soil-grown plants were harvested after the shift to LD. For RNA- and ChIP-seq analysis, ethanol-sterilized seeds were grown on 1× MS (Murashige and Skoog) medium with glucose at LD conditions in a Percival incubator for 10 days. All seeds were incubated at 4 °C in the dark for 3–5 days prior to germination.

### Cloning and transgenic lines

Genetic backgrounds of transgenic lines used in this study are listed in Additional file 1: Table S2. A CRISPR/Cas9 vector pKIR1.0 [52] containing tandem sgRNA cassettes for HTR4 and HTR5 was used to generate *htr4;htr5* double mutants. Target sequences for HTR4 and HTR5 were 5′-gCCTCCGGTGGACTTACGAG-3′ and 5′-gCAGCTCGTAAGTCTACTGG-3′, respectively. Cas9-free T2 seeds were selected by absence of seed fluorescence, and T2 plants were screened by direct sequencing of HTR4 and HTR5 gene loci to obtain *htr4;htr5* double homozygous mutants.

Cloning was done using the Gateway® Cloning Technology (Invitrogen) or PCR-based site-directed mutagenesis. The artificial miRNAs (amiRNAs) *amiR-HTR5-I* and *amiR-HTR5-II* were designed and cloned according to the original protocol [53], with minor modifications. Sequences of the amiRNAs can be found in Additional file 2: Figure S2. Primers are listed in Additional file 1: Table S5. Briefly, primers were combined in three independent PCR reactions using M13-fwd and M13-rev primers instead of primers A and B of the original protocol (http://wmd3.weigelworld.org). PCR products were purified (QIAquick PCR Purification Kit, Qiagen) and combined in a fusion PCR using primers designed to add *att*B1/*att*B2 tails. Resulting amiRNAs were recombined using the Gateway® BP Clonase^TM^ II enzyme into pDONR^TM^221 and used for subsequent Multisite Gateway® recombination with the LR Clonase^TM^ II Plus enzyme (all Invitrogen) into a modified, Multisite Gateway® compatible pAlligator-MGW binary plasmid, under control of the *HTR5* promoter. The *HTR5* promoter [14] was amplified adding *att*B4/*att*B1r sites and recombined into pDONR^TM^ P4-P1R (Invitrogen). For the third cassette in the Multisite Gateway® system a short nucleotide sequence was designed and recombined with *att*B2r/*att*B3 sites into pDONR^TM^ P2R-P3 (Invitrogen), as “empty plasmid”. Resulting binary plasmids *pHTR5-amiR-HTR5-I* (pHW358) and *pHTR5-amiR-HTR5-II* (pHW359) were transformed into plants descending from a *htr4-2/+;htr8-2* parent, and therefore segregating for the *htr4-2* T-DNA insertion, using a simplified floral dip method. Primary transgenic plants were selected by green fluorescence in dry seeds, grown, and phenotypically characterized. For each transgene a subset of lines with single T-DNA insertion was followed into subsequent generations.

The *HTR5* gene was amplified and cloned into pDONR^TM^221 as described above. Two independent steps of site-directed mutagenesis were performed on this plasmid to introduce silent mutations, rendering the resulting *rHTR5* transcript resistant against *amiRNA-HTR5-II* targeting and to introduce a STOP codon. Multisite Gateway® recombination resulted in the final binary plasmid *pHTR5-rHTR5* (pHW375) in pSRR4R3-19ST, which were transformed into *htr4-2*;*htr8-2* double homozygous plants. Primary transgenic lines were selected by red fluorescence in dry seeds, phenotypically characterized, and, for a subset of lines, followed into subsequent generations. F1 seeds resulting from crosses to *h3.3kd-3* were confirmed by green and red fluorescence in dry seeds. Phenotypic analysis of rosette size, leaf serration, and silique length was carried out in F1 plants. Plant genotypes are listed in Additional file 1: Table S2.

### Immunofluorescence

Immunofluorescence experiments were performed as described previously [54] on isolated nuclei from mature leaves of *h3.3kd-1* (not shown) and *h3.3kd-2* plants (Fig. 2a), with similar results. We used antibodies from Abcam (H3K9me2, ab1220; H3K4me3, ab8580). The H3K27me3 antibody was a generous gift from Thomas Jenuewein, Max Planck Institute of Immunobiology and Epigenetics in Freiburg, Germany.

### RNA-seq and microarray analysis

RNA was extracted from pooled 10-day-old seedlings grown on 1× MS medium with glucose as described above, using the RNeasy Mini Kit (Qiagen). Concentration and purity were determined with Nanodrop measurement and, dependent on the values, total RNA extracts were subjected to additional ethanol precipitation or used directly for subsequent steps. For RNA-seq, total RNA was sent to AITbiotech (Singapore) for library generation, sequencing on the Illumina platform, and subsequent data analysis. Briefly, after mRNA enrichment and fragmentation to ~200-bp fragments, first strand cDNA synthesis with random hexamer-primers and second strand cDNA synthesis, cDNA was purified with a QIAquick PCR purification kit (Qiagen). After end repair and A-tailing, sequencing adaptors were ligated to the fragments; fragments were purified on agarose gel, PCR amplified, and sequenced on a HiSeq^TM^2000 (Illumina). TopHat, cufflinks, cuffmerge, and cuffdiff were used for analysis, TAIR9 was used for alignment, and expression values are FPKM values. Gene Ontology analysis was performed using DAVID (http://david.abcc.ncifcrf.gov/) [55, 56]. A single dataset was obtained and results were confirmed based on additional analyses of microarrays (Additional file 2: Figure S2e). We performed technical duplicates using 10-day-old plate-grown seedlings of *h3.3kd* (*htr4;htr8;amiRHTR5-II*) and *h3.3kd/+* (*htr4;htr8;amiRHTR5-II/+*). For microarray analysis, total RNA extracted from 10-day-old seedlings by an RNeasy Mini Kit (Qiagen) was hybridized to an ATH1(Affymetrix) array according to the manufacturer’s instructions. Biotin was used to label extracts. Fragmentation, hybridization, washing, and scanning were performed at BSF (Singapore). All datasets were normalized by log2GC-RMA (Robust Multichip Average) methods.

The specific enrichment in hypervariable genes affected by the loss of H3.3 was statistically significant [57]. We applied the hypergeometric test as follows: population size, 21340 (expressed genes in Col-0, FPKM >0.5 in the RNA-Seq); number of successes in population, 123 (all hypervariable genes); sample size, 669 (downregulated genes excluding *HTR4*, *5*, and *8*); number of successes in sample, 44 (overlap downregulated genes and hypervariable genes). Result is *p*(X ≥ 44) = 8.e-35.

### ChIP-seq analysis

ChIP was also performed on 10-day-old plate-grown seedlings and according to a previously described procedure [19]. The following commercially available antibodies were used: H3 (Abcam, ab1791), H3K4me3 (Abcam, ab8580), H3K36me3 (Abcam, ab9050), and H1 (Agrisera, AS11 1801). The H2A.Z antibody was generated in our laboratory and has been described elsewhere [54]. ChIP-seq libraries were generated according to the manufacturer’s instructions and sequenced on a HiSeq2000 (Illumina). Reads were mapped to the TAIR10 genome by allowing up to two mismatches and only retaining reads that map uniquely to the genome using Bowtie [58]. Reads mapping to the same coordinates were removed, and data were normalized by total number of uniquely mapping reads. For the metaplots, flanking regions were scaled to the same length as the gene body (middle region). Duplicates were obtained for all datasets.

### Methylation analysis

Mature leaves from Col-0 WT and *h3.3kd-2* and *h3.3kd-3* were collected and ground in liquid nitrogen to a fine powder. DNA was extracted using the DNeasy Plant Mini Kit (Qiagen) according to the manufacturer’s instructions and Bi-seq and data analysis were carried out as previously described [47]. Briefly, identical reads were collapsed into one read, and reads were mapped to the TAIR10 genome using BS seeker [59] and methylation levels were calculated as #C/(#C + #T). For the metaplots, flanking regions were scaled to the same length as the gene body (middle region). Differentially methylated regions (DMRs) in *h3.3kd* were defined with a method similar to a previously published DMR-finding approach [47]. The genome was binned into 100-bp tiles, where tiles with less than 20 C + T calls in any sample were omitted, and tiles with Benjamini–Hochberg-corrected FDR <0.01 (Fisher’s exact test) and a 20% absolute methylation reduction were selected. Only tiles that met these criteria in two biological replicates of *h3.3kd* data were retained and finally tiles within 200 bp of each other were merged.

## Additional files

Additional file 1: Supplemental tables. **Table S1** Summary of H3.3 T-DNA insertion lines. **Table S2** Plant genetic backgrounds. **Table S3** Expression of selected chromatin factors in WT and *h3.3kd*. **Table S4** Limited overlap between H3.3 enrichment in WT and transcriptional changes in *h3.3kd*. **Table S5** Primer sequences. (DOCX 36 kb)

Additional file 2: Supplemental figures. **Figure S1** Generation of *h3.3KO.* **Figure S2** Generation of *h3.3kd.* **Figure S3** Impact of *h3.3kd* on active chromatin modifications. (DOCX 1094 kb)

Additional file 3: List of genes significantly downregulated in *h3.3kd-3* compared to WT. (XLS 171 kb)

Additional file 4: List of genes significantly upregulated in *h3.3kd-3* compared to WT. (XLS 116 kb)

Additional file 5: GO term categories of significantly downregulated genes in *h3.3kd-3* compared to WT. (XLS 86 kb)

Additional file 6: List of hypomethylated TEs in *h3.3kd-3*. (XLS 190 kb)
