# Supplementary material for: The histone H3 variant H3.3 regulates gene body DNA methylation in Arabidopsis thaliana
Source: Genome Biol. 2017 May 18;18:94. doi: 10.1186/s13059-017-1221-3 (PMC5437678; doi:10.1186/s13059-017-1221-3)
Supplement: Supplementary file 1 — Supplemental tables. Table S1 Summary of H3.3 T-DNA insertion lines. Table S2 Plant genetic backgrounds. Table S3 Expression of selected chromatin factors in WT and h3.3kd. Table S4 Limited overlap between H3.3 enrichment in WT and transcriptional changes in h3.3kd. Table S5 Primer sequences. (DOCX 36 kb) [file 13059_2017_1221_MOESM1_ESM.docx]

**The histone H3 variant H3.3 regulates gene body DNA methylation in *Arabidopsis thaliana***

**Additional file 1: Supplemental Tables**

Supplemental Table 1: Summary of H3.3 T-DNA insertion lines.

| **Gene** | **ID** | **T-DNA line** | **Name** | **Leaf phenotype** |
| --- | --- | --- | --- | --- |
| *HTR4* | At4g40030 | N582765 ^a,b^ | *htr4-1* | wildtype-like |
|  |  | N807939 ^b^ | *htr4-2* | wildtype-like |
| *HTR5* | At4g40040 | N510583 ^a^ | *htr5-1* | wildtype-like |
|  |  | N808947 | *htr5-2* | wildtype-like |
|  |  | N846395 ^b^ | *htr5-3* | wildtype-like |
| *HTR8* | At5g10980 | N587850 ^a^ | *htr8-1* | wildtype-like |
|  |  | N641101 ^b^ | *htr8-2* | wildtype-like |

^a^[32], ^b^ this work

Supplemental Table 2: Plant genetic backgrounds.

| **Name** | **T-DNA / CRISPR/Cas9** | **Transgene** | **Description** |
| --- | --- | --- | --- |
| *h3.3KO* | *htr4;htr5;htr8-2* | none | *H3.3* knockout |
| *h3.3kd-1* | *htr4-2;htr8-2* | *amiR-HTR5-I* | *H3.3* knockdown |
| *h3.3kd-2, -3* | *htr4-2;htr8-2* | *amiR-HTR5-II* | *H3.3* knockdown |
| *h3.3kd/+* | *htr4-2;htr8-2* | *amiR-HTR5-II/+* | F1 cross of *h3.3kd-3*, hemizygous transgene |
| *rH3.3* | *htr4-2;htr8-2* | *rHTR5* | *amiR-HTR5-II* resistant *rHTR5* |
| *h3.3kd/+;rH3.3/+* | *htr4-2;htr8-2* | *amiR-HTR5-II/+, rHTR5/+* | F1 cross of *h3.3kd-3* and *rH3.3*, both transgenes hemizygous |

Supplemental Table 3: Expression of selected chromatin factors in WT and *h3.3kd*.

| **Category** | **Gene** | **Gene ID** | **WT** | ***h3.3kd*** | **log2(FC)** | **p_value** | **q_value** |
| --- | --- | --- | --- | --- | --- | --- | --- |
| Potential H3 chaperone | *HIRA* | AT3G44530 | 3.83 | 4.86 | 0.34 | 0.18 | 0.68 |
|  | *ATRX* | AT1G08600 | 11.56 | 12.11 | 0.07 | 0.74 | 0.97 |
|  | *ASF1A* | AT1G66740 | 16.49 | 14.52 | -0.18 | 0.49 | 0.91 |
|  | *ASF1B* | AT5G38110 | 10.29 | 13.26 | 0.37 | 0.23 | 0.74 |
|  | *MSI1* | AT5G58230 | 20.06 | 24.37 | 0.28 | 0.20 | 0.70 |
|  | *FAS1* | AT1G65470 | 6.79 | 6.58 | -0.05 | 0.86 | 0.98 |
|  | *FAS2* | AT5G64630 | 3.81 | 4.58 | 0.27 | 0.28 | 0.79 |
| Potential H2A.Z chaperone | *ARP6* | AT3G33520 | 23.94 | 22.39 | -0.10 | 0.66 | 0.95 |
|  | *PIE1* | AT3G12810 | 6.67 | 7.81 | 0.23 | 0.27 | 0.77 |
|  | *SEF* | AT5G37055 | 17.34 | 16.53 | -0.07 | 0.83 | 0.98 |
| DNA methylation related | *METI* | AT5G49160 | 12.32 | 11.62 | -0.08 | 0.68 | 0.96 |
|  | *DRM2* | AT5G14620 | 10.33 | 9.38 | -0.14 | 0.56 | 0.93 |
|  | *DRM3* | AT3G17310 | 10.02 | 10.13 | 0.02 | 0.92 | 0.99 |
|  | *CMT2* | AT4G19020 | 7.59 | 7.34 | -0.05 | 0.83 | 0.98 |
|  | *CMT3* | AT1G69770 | 11.69 | 12.46 | 0.09 | 0.67 | 0.96 |
|  | *SUVH4* | AT5G13960 | 6.02 | 5.58 | -0.11 | 0.69 | 0.96 |
|  | *SUVH5* | AT2G35160 | 2.17 | 3.34 | 0.62 | 0.06 | 0.44 |
|  | *SUVH6* | AT2G22740 | 7.37 | 7.16 | -0.04 | 0.87 | 0.98 |
|  | *DDM1* | AT5G66750 | 5.20 | 5.76 | 0.15 | 0.58 | 0.94 |
|  | *ROS1/DML1* | AT2G36490 | 17.79 | 12.40 | -0.52 | 0.01 | 0.14 |
|  | *RPA32A* | AT2G24490 | 8.13 | 11.80 | 0.54 | 0.08 | 0.48 |
|  | *DME* | AT5G04560 | 22.02 | 19.30 | -0.19 | 0.33 | 0.83 |
|  | *DML2* | AT3G10010 | 5.81 | 5.44 | -0.10 | 0.68 | 0.96 |
|  | *DML3* | AT4G34060 | 0.82 | 0.49 | -0.74 | 0.14 | 0.63 |
|  | *IBM1* | AT3G07610 | 8.84 | 7.25 | -0.29 | 0.16 | 0.65 |
|  | *SHH1* | AT1G15215 | 10.75 | 9.96 | -0.11 | 0.68 | 0.96 |
|  | *SDC* | AT2G17690 | 0.21 | 0.97 | 2.18 | 0.00 | 0.09 |
| Histone H1 | *HON1* | AT1G06760 | 226.36 | 299.03 | 0.40 | 0.05 | 0.37 |
|  | ***HON2*** | **AT2G30620** | **167.00** | **272.96** | **0.71** | **0.00** | **0.02** |
|  | ***HON3*** | **AT2G18050** | **25.09** | **231.05** | **3.20** | **0.00** | **0.00** |
| Histone H2A | *HTA1* | AT5G54640 | 37.31 | 41.34 | 0.15 | 0.54 | 0.93 |
|  | *HTA2* | AT4G27230 | 40.64 | 49.73 | 0.29 | 0.21 | 0.72 |
|  | *HTA10* | AT1G51060 | 96.15 | 119.95 | 0.32 | 0.12 | 0.58 |
|  | *HTA13* | AT3G20670 | 27.52 | 35.67 | 0.37 | 0.16 | 0.64 |
|  | *HTA3* | AT1G54690 | 45.62 | 61.73 | 0.44 | 0.05 | 0.36 |
|  | *HTA5* | AT1G08880 | 118.90 | 130.04 | 0.13 | 0.52 | 0.92 |
|  | *HTA6* | AT5G59870 | 58.41 | 81.57 | 0.48 | 0.02 | 0.24 |
|  | ***HTA7*** | **AT5G27670** | **54.10** | **90.96** | **0.75** | **0.00** | **0.01** |
|  | *HTA12* | AT5G02560 | 13.34 | 15.32 | 0.20 | 0.56 | 0.93 |
|  | *HTA8* | AT2G38810 | 13.11 | 14.18 | 0.11 | 0.69 | 0.96 |
|  | *HTA9* | AT1G52740 | 133.18 | 110.22 | -0.27 | 0.17 | 0.66 |
|  | *HTA11* | AT3G54560 | 38.77 | 41.66 | 0.10 | 0.67 | 0.96 |
|  | *HTA4* | AT4G13570 | 5.94 | 5.22 | -0.18 | 0.77 | 0.97 |
| Histone H2B | *HTB1* | AT1G07790 | 40.66 | 54.21 | 0.41 | 0.07 | 0.45 |
|  | *HTB2* | AT5G22880 | 151.69 | 189.51 | 0.32 | 0.10 | 0.55 |
|  | *HTB3* | AT2G28720 | 137.08 | 174.74 | 0.35 | 0.08 | 0.48 |
|  | *HTB4* | AT5G59910 | 181.31 | 196.89 | 0.12 | 0.54 | 0.93 |
|  | *HTB5* | AT2G37470 | 51.99 | 68.18 | 0.39 | 0.09 | 0.51 |
|  | ***HTB6*** | **AT3G53650** | **8.31** | **33.70** | **2.02** | **0.00** | **0.00** |
|  | *HTB7* | AT3G09480 | 5.40 | 8.94 | 0.73 | 0.11 | 0.55 |
|  | *HTB8* | AT1G08170 | 0.00 | 0.00 | 0.00 | 1.00 | 1.00 |
|  | *HTB9* | AT3G45980 | 183.58 | 268.07 | 0.55 | 0.01 | 0.10 |
|  | *HTB10* | AT5G02570 | 1.63 | 3.20 | 0.97 | 0.22 | 0.73 |
|  | *HTB11* | AT3G46030 | 158.87 | 201.37 | 0.34 | 0.08 | 0.49 |
| Histone H3 | ***HTR1*** | **AT5G65360** | **119.02** | **205.75** | **0.79** | **0.00** | **0.00** |
|  | ***HTR2*** | **AT1G09200** | **154.17** | **272.70** | **0.82** | **0.00** | **0.00** |
|  | ***HTR3*** | **AT3G27360** | **20.81** | **48.81** | **1.23** | **0.00** | **0.00** |
|  | *HTR9* | AT5G10400 | 48.68 | 58.86 | 0.27 | 0.23 | 0.74 |
|  | *HTR13* | AT5G10390 | 18.08 | 29.26 | 0.69 | 0.01 | 0.18 |
|  | ***HTR4*** | **AT4G40030** | **336.33** | **79.26** | **-2.09** | **0.00** | **0.00** |
|  | ***HTR5*** | **AT4G40040** | **426.33** | **197.87** | **-1.11** | **0.00** | **0.00** |
|  | ***HTR8*** | **AT5G10980** | **208.31** | **9.62** | **-4.44** | **0.00** | **0.00** |
|  | *HTR6* | AT1G13370 | 0.10 | 0.50 | 2.30 | 0.15 | 0.63 |
|  | *HTR10* | AT1G19890 | 0.00 | 0.00 | 0.00 | 1.00 | 1.00 |
|  | *HTR14* | AT1G75600 | 0.14 | 0.27 | 0.97 | 0.58 | 1.00 |
|  | *HTR12* | AT1G01370 | 4.61 | 4.64 | 0.01 | 0.98 | 1.00 |
|  | *HTR11* | AT5G65350 | 1.22 | 2.50 | 1.04 | 0.12 | 0.59 |
|  | *HTR15* | AT5G12910 | 0.00 | 0.00 | 0.00 | 1.00 | 1.00 |
|  | *HTR7* | AT1G75610 | 0.00 | 0.47 | 1.79769E+308 | 0.02 | 0.24 |
| Histone H4 | ***HF01*** | **AT3G46320** | **26.76** | **51.27** | **0.94** | **0.00** | **0.03** |
|  | ***HF02*** | **AT5G59690** | **93.17** | **210.59** | **1.18** | **0.00** | **0.00** |
|  | ***HF03*** | **AT2G28740** | **73.05** | **175.16** | **1.26** | **0.00** | **0.00** |
|  | ***HF04*** | **AT1G07820** | **76.76** | **164.69** | **1.10** | **0.00** | **0.00** |
|  | ***HF05*** | **AT3G53730** | **116.10** | **505.44** | **2.12** | **0.00** | **0.00** |
|  | ***HF06*** | **AT5G59970** | **41.24** | **88.68** | **1.10** | **0.00** | **0.00** |
|  | ***HF07*** | **AT3G45930** | **30.40** | **63.46** | **1.06** | **0.00** | **0.00** |
|  | *HF08* | AT1G07660 | 30.96 | 51.18 | 0.73 | 0.00 | 0.06 |

Supplemental Table 4: Limited overlap between H3.3 enrichment in WT and transcriptional changes in *h3.3kd*.

|  |  | **Overlap with misregulated genes** | |
| --- | --- | --- | --- |
| **Location of H3.3 enrichment** | **Number of genes*** | **Downregulated (N=661)** | **Upregulated (N=269)** |
| promoter-only  (TSS +/-500 bp) | 983 | 26 (2.6%) | 16 (1.6%) |
| gene body-only  (TSS +500bp to TTS) | 9,035 | 175 (1.9%) | 71 (0.8%) |
| promoter and gene body | 9,860 | 283 (2.9%) | 133 (1.3%) |

*Overlap of all genes >500bp (N= 25,390) and defined H3.3 regions [18]

Supplemental Table 5: Primer sequences.

| **Purpose** | **Primer** | **Sequence** |
| --- | --- | --- |
| Cloning *amiR-HTR5-I* | I miR-s | gaTATCTATTTAGAAAACCGCCCtctctcttttgtattcc |
|  | II miR-as | gaGGGCGGTTTTCTAAATAGATAtcaaagagaatcaatga |
|  | III miR*-s | gaGGACGGTTTTCTATATAGATTtcacaggtcgtgatatg |
|  | IV miR*-as | gaAATCTATATAGAAAACCGTCCtctacatatatattcct |
| Cloning *amiR-HTR5-II* | I miR-s | gaTTCCAGTAGACTTACGCGCTGtctctcttttgtattcc |
|  | II miR-as | gaCAGCGCGTAAGTCTACTGGAAtcaaagagaatcaatga |
|  | III miR*-s | gaCAACGCGTAAGTCAACTGGATtcacaggtcgtgatatg |
|  | IV miR*-as | gaATCCAGTTGACTTACGCGTTGtctacatatatattcct |
| amiR fusion with *att*B | *attB1*-A | GGGGACAAGTTTGTACAAAAAAGCAGGCTCGATAAGCTTGATATCGAATTCCT |
|  | *attB2*-B | GGGGACCACTTTGTACAAGAAAGCTGGGTGGCCGCTCTAGAACTAGTGGA |
| Cloning *HTR5* | *HTR5*-fwd | GGGGACAAGTTTGTACAAAAAAGCAGGCTGTATGGCTCGTACTAAGCAAACA |
|  | *HTR5*-rev | GGGGACCACTTTGTACAAGAAAGCTGGGTTAGCACGTTCTCCTCTGATCCTG |
| Mutagenesis *rHTR5* | *rHTR5*-fwd | CTAAGCAAACAGCTCGTAAAAGCACAGGAGGAAAGGCTCCTAGG |
|  | *rHTR5*-rev | CCTAGGAGCCTTTCCTCCTGTGCTTTTACGAGCTGTTTGCTTAG |
| Mutagenesis *rHTR5-STOP* | *STOP*-fwd | GAGGAGAACGTGCTTAAAACCCAGCTTTCTTG |
|  | *STOP*-rev | CAAGAAAGCTGGGTTTTAAGCACGTTCTCCTC |
| RT-PCR | *HTR4* | TCCCCTATTACTGCTTTTGTTACG |
|  |  | AAATCTCGGATTCTTGGTTGG |
|  | *HTR5* | TTTTCGAATTCGGAGCTCAA |
|  |  | AGAAAACCACCCACATTCACA |
|  | *HTR8* | CGAAAAGTCACATCTTTTGGAA |
|  |  | GCGATAGGAAACAAGAATCCA |
