# Supplementary material for: The histone H3 variant H3.3 regulates gene body DNA methylation in Arabidopsis thaliana
Source: Genome Biol. 2017 May 18;18:94. doi: 10.1186/s13059-017-1221-3 (PMC5437678; doi:10.1186/s13059-017-1221-3)
Supplement: Supplementary file 2 — Supplemental figures. Figure S1 Generation of h3.3KO. Figure S2 Generation of h3.3kd. Figure S3 Impact of h3.3kd on active chromatin modifications. (DOCX 1094 kb) [file 13059_2017_1221_MOESM2_ESM.docx]

**Additional file2: Supplemental Figures**

**
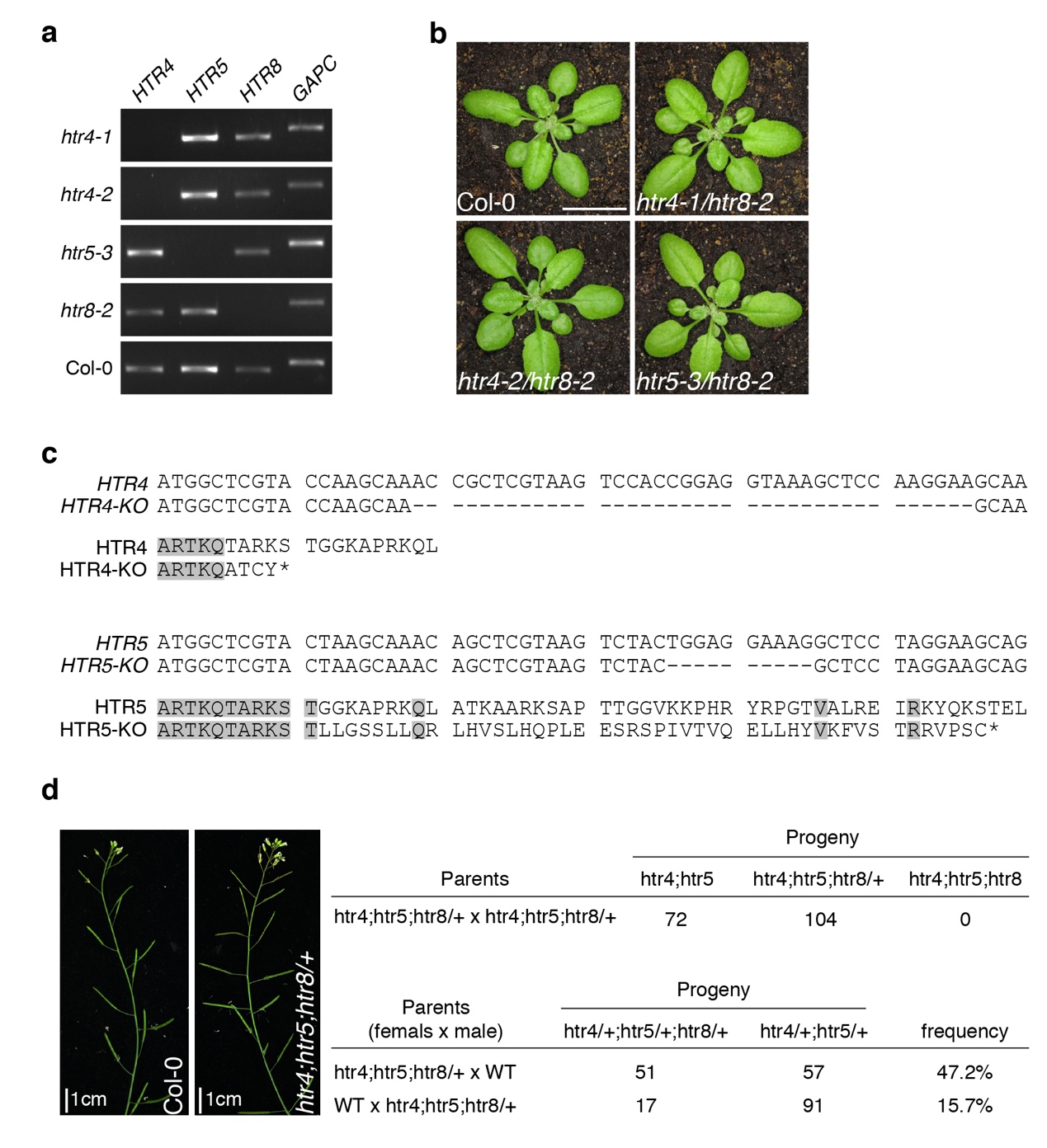
**

**Additional file 2 Figure S1: Generation of *h3.3KO*.**

(**a**) RT-PCR analysis of full-length transcript (5’ to 3’ UTR) in *H3.3* T-DNA insertion lines. (**b**) Phenotypes of three different *H3.3* double mutant combinations compared to WT. (**c**) Partial gene and protein sequence of HTR4 and HTR5 in wildtype and *htr4;htr5* CRISPR/Cas9 double mutant. (**d**) Phenotype of Col-0 wildtype and *htr4;htr5;htr8/+* siliques and frequencies of progenies obtained from crosses.


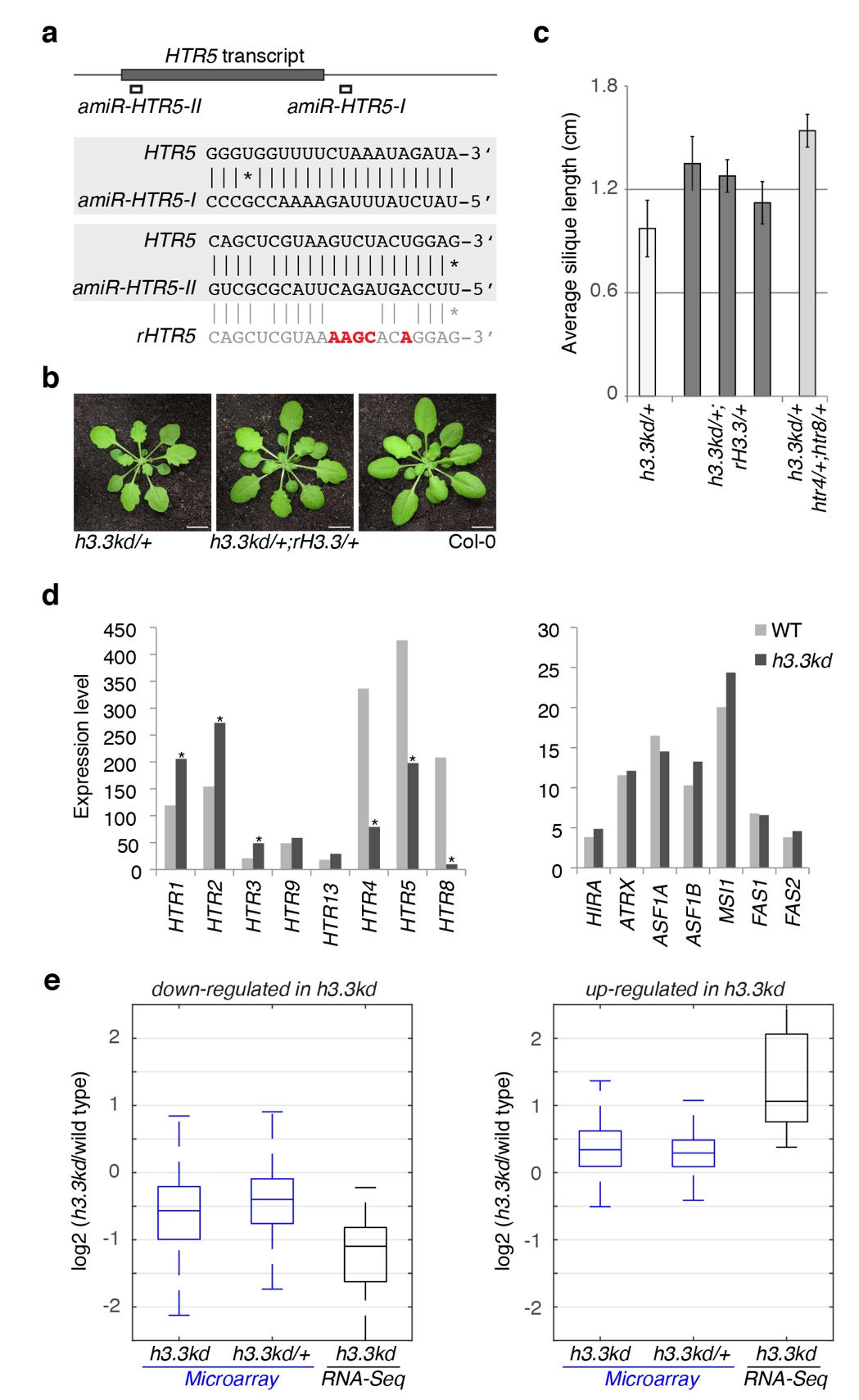


**Additional file 2 Figure S2: Generation of *h3.3kd*.**

(**a**) Sequences of the two artificial miRNAs *amiR-HTR5-I* and *amiR-HTR5-II* designed to target two different regions of the *HTR5* transcript (shown schematically on top). Design of *rHTR5* is shown at the bottom. Silent mutations within the *amiR-HTR5-II* target site ensure limited complementarity to the amiRNA. (**b, c**) Phenotypic rescue of *h3.3kd*-specific serrated leaf shape and smaller rosette size. Compared with *h3.3kd/+*, i.e. *htr4-2:htr8-2* double mutants with only one copy of the *amiR-HTR5-II* transgene (*htr4;htr8;amiR-HTR5-II/+*, left panel), a partial rescue of the reduced growth and leaf shape (**b**) and sterility measure by silique elongation (**c**) is observed in *h3.3kd/+;rH3.3/+*, i.e. *htr4-2:htr8-2* double mutants with one copy of the *amiR-HTR5-II* transgene and one copy of the *rHTR5* amiR-resistant *HTR5* transgene (*htr4;htr8;amiR-HTR5-II/+;rHTR5/+*, middle panel); three independent transgenic *rH3.3* lines. Average values reflect 10 siliques per plant from three or more plants for each genotype. (**d**) Expression levels of histone H3 variants and putative H3 chaperones from RNA-seq analysis of WT and *h3.3kd*. (**e**) Misexpression of genes in *h3.3kd* versus WT Col, downregulated genes (left panel) and upregulated genes (right panel). Trends from genes misexpressed in RNA-Seq profiles (black) are similar to microarray-based profiles (blue).


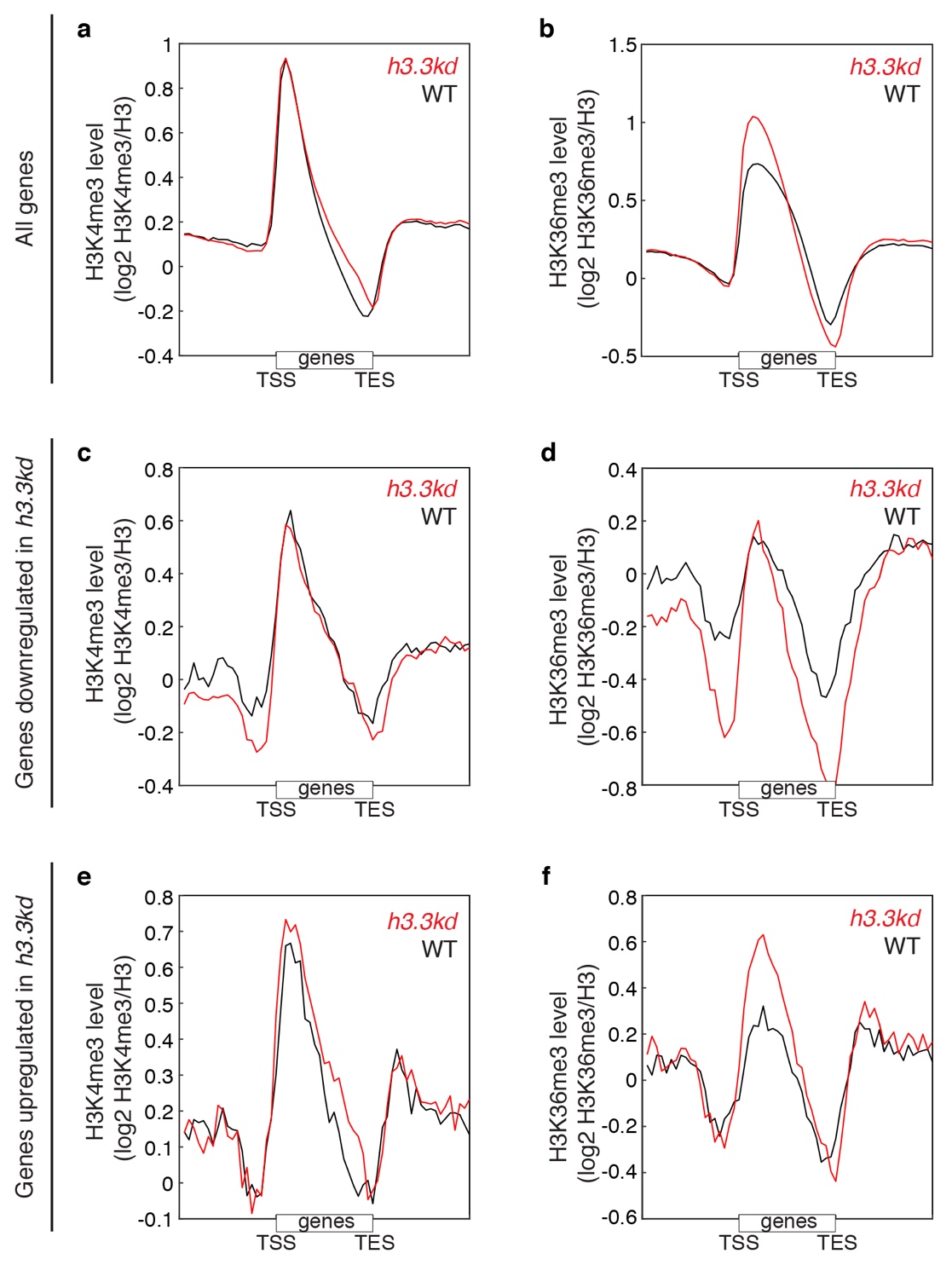


**Additional file 2 Figure S3: Impact of *h3.3kd* on active chromatin modifications.**

(**a**) ChIP-seq profiles depicting the enrichment of H3K4me3 in WT and *h3.3kd* over all genes. (**b**) ChIP-seq profiles depicting the enrichment of H3K36me3 in WT and *h3.3kd* over all genes. (**c, d**) ChIP-seq profiles depicting the enrichment of H3K4me3 (**c**) and H3K36me3 (**d**) in WT and *h3.3kd* over genes downregulated in *h3.3kd*. (**e, f**) ChIP-seq profiles depicting the enrichment of H3K4me3 (**e**) and H3K36me3 (**f**) in WT and *h3.3kd* over genes upregulated in *h3.3kd*.
